# The dynamics of decision-making in weight loss and maintenance: a qualitative enquiry

**DOI:** 10.1186/s12889-020-08664-y

**Published:** 2020-04-28

**Authors:** Leon Poltawski, Samantha Barbara van Beurden, Sarah Morgan-Trimmer, Colin Greaves

**Affiliations:** 1 University of Exeter Medical School, St Luke’s, Heavitree Rd, Exeter, EX1 2LU UK; 2 School of Psychological Science, University of Bristol, Priory Road Complex, Priory Road, Bristol, BS8 1TU UK; 3 School of Sport, Exercise & Rehabilitation Sciences, University of Birmingham, Edgbaston, Birmingham B15 2TT UK

**Keywords:** Weight management, Mechanisms, Maintenance, Behaviour change, Qualitative

## Abstract

**Background:**

Behavioural approaches to weight loss are often initially successful but less so in the longer term, as some people maintain the necessary behaviour changes while others do not. This study aimed to derive possible explanations for this using a qualitative approach with a view to improving intervention effectiveness.

**Methods:**

Thirty-six participants in a development and feasibility study for a weight loss and maintenance intervention (called SkiM) were interviewed three times over 18 months regarding their experiences before, during and after the intervention. Data were analysed thematically. The accounts of those who were more and less successful in terms of longer term weight loss were compared, and a conceptual model linking the main analytic themes was developed.

**Results:**

Five interpretative themes were generated: encountering and managing key situations; the impact of emotion; the source of control; personal values; and acquiring knowledge and skills. These themes were linked through a model of decision-making during key situations. In this model, behavioural decisions emerge from a dynamic interplay between several drivers: emotional state and needs, perceived control, personal values, the individual’s knowledge and skills, and their existing habits. The individual’s response in key situations generates experiential learning that can influence decisional dynamics in similar situations in future. These dynamics appeared to differ between participants, and between those who were more and less successful in weight management.

**Conclusions:**

Our analysis and model of decision-making during weight-management have implications for the development and delivery of behavioural weight management interventions. By helping individuals to identify the drivers of their decision-making in key situations, and equipping them to manage these drivers, programmes may enhance their capacity to sustain the behaviour changes needed for long-term weight loss.

## Background

Obesity continues to be a global and growing problem because of its impact upon individual health and well-being, and the costs of treating the illnesses it can cause [1]. Various weight management interventions ranging from educational to surgical are available, and these can result in substantial initial weight loss [2–4]. However weight regain is common [5]. Moreover, trials often report large variance in outcomes, with some participants achieving and maintaining substantial weight loss while others lose little or no weight. The reasons for this are complex and involve interrelated physiological, psychological and sociological factors [6]. While studies are teasing out some of the underlying mechanisms [7–10], there is still much to be learned about how they operate at an individual level, and why some people are more successful than others in both losing weight and maintaining weight loss [11, 12].

Qualitative studies can provide valuable in-depth data to increase understanding of these mechanisms. Where they are used in evaluations of interventions, they can help identify factors and processes that influence outcomes [13] and suggest reasons for heterogeneity in those outcomes [14]. For example, one weight management study [15] confirmed the importance of supplementary human support in an online weight loss intervention, but also helped explain why participants might not avail of such support. In another interventional study [16], qualitative data suggested that differences in outcomes depended partly on the type and extent of problem-solving skills employed by participants. These studies illustrate the value of qualitative research that focuses on the mechanisms of behaviour change. They can deepen our understanding of processes underpinning change, and suggest ways of fine-tuning interventions to more effectively target those processes.

We conducted a qualitative enquiry as part of the development and feasibility study of a behavioural intervention for weight management, called ‘Skills for weight loss and Maintenance (SkiM)’. Full descriptions of the SkiM programme, its development and its evaluation, are available elsewhere (Greaves et al., under review). Briefly, the programme comprised a series of 90-min group-based workshops over 6 months, led by two trained facilitators. As part of the programme development process, two versions were offered, one with thirteen workshops and the other with six workshops and three one-to-one consultations, two of which were optional. Both programme versions covered the same topics but in different levels of detail. Each group meeting involved a review of experience, topic-based discussion and activities, and skills training for weight-related behaviour change. Topics included nutrition and physical activity for weight management, problem-solving, and principles of sustainable behaviour change. Skills training addressed self-regulation, for example planning and self-monitoring, and identifying and managing the personal and external factors that influence behaviour. An important principle of the SkiM approach was that, although facilitators provided advice and support, decisions on the selection of personal strategies for weight management were left to the individual. This was intended to encourage personal ownership of the endeavour, and increase the likely sustainability of behaviour changes.

The development and feasibility study aimed to evaluate and refine the SkiM programme, and data were collected from each participant before, during and after the intervention, over an 18 month period. The SkiM programme was delivered to an initial cohort of participants, then refined on the basis of data and opinions collected from participants, before being delivered and evaluated with a second cohort of participants. Within this broader study, we conducted a qualitative investigation with a sub-sample of participants, to address the question: what factors may influence individual experiences and outcomes in the SkiM weight management programme? By enhancing our understanding of why some people did better than others, we hoped to identify potential improvements in the intervention. This paper reports that investigation, and follows the guidelines provided in the COREQ consolidated criteria for reporting qualitative studies [17]. The completed checklist is provided in Additional file 1.

## Methods

The SkiM development and feasibility study, including this qualitative component, was registered with the ISRCTN (ID 45134679) (10.1186/ISRCTN45134679), reviewed and approved by the local Research Ethics Committee of the UK Health Research Authority, and was conducted in the UK between August 2015 and May 2018. All participants gave written informed consent.

### Participants

Eligible participants in the main SkiM study were over 18 years old, had a body mass index (BMI) ≥ 30 kg/m^2^ (or 27.5 kg/m^2^ for those of African/Caribbean or south Asian heritage), were willing to make lifestyle changes for weight loss, and were able to communicate in English. Recruitment used two routes: (i) letters sent by General Practitioners (GPs) to a random sample of their patients meeting age and BMI criteria, or (ii) verbal invitations issued by the staff of National Health Service (NHS) healthy lifestyle hubs to individuals who had been referred by their GPs for signposting to weight management services. The main study began with 100 participants, all of whom were interviewed at baseline, which enabled sub-sampling for follow-up interviews based on weight loss during the programme. After the intervention, 36 participants were purposively selected for the qualitative sub-study, ensuring inclusion of those who were successful in initial weight loss and those who were not, men and women, and the age range of the full sample. Those selected for this study were interviewed within 4 weeks of the programme ending (~ 6 months post-baseline), and again 12 months later (18 months post-baseline). Some participants withdrew from the main study before the intervention ended and were not included in the sampling frame, but all of those invited to follow-up interviews agreed to participate.

### Data collection

Demographic, weight, and programme attendance data were collected as part of the main study (collection and analysis procedures are reported elsewhere: Greaves et al., under review). Baseline interviews were conducted by either LP or SvB (see Additional file 1 for interviewer characteristics and training). All follow-up interviews were conducted by LP. Interviews were face-to-face, either at the researchers’ University or at the participant’s home, according to their preference. They were audio-recorded digitally and transcribed verbatim for analysis, and field notes were made by the interviewer.

All interviews were semi-structured, using topic guides formulated specifically for this study to address the aims of the broader feasibility study and of this nested investigation. At baseline, participants were asked about their weight management history and reasons for joining, and expectations of, the programme. These data were used to inform subsequent development of the programme. Follow-up interviews focussed on their weight management experience, although the second interview was also concerned with evaluation of the feasibility and acceptability of the SkiM programme (which is reported elsewhere by the authors; Greaves et al., under review). Additional file 2 provides the full topic guides, but the main questions relating to this study were:

- Why do you want to lose weight?
- What have been the main influences on your weight?
- What have you done to help manage your weight?
- What has helped and hindered you in managing your weight?
- How do you feel about the weight management process?

### Analysis

Thematic analysis, drawing on principles outlined by Marks and Yardley [18] and Braun and Clarke [19], was employed to explore participants’ accounts of their weight management experiences. Thematic analysis was selected because of its flexibility: it can facilitate development of both structured descriptions and conceptual models, while identifying areas of commonality and divergence across a sample. These features were important for the analysis, because its purpose was to describe the experiences of a diverse group of individuals, and to identify potential mechanisms linking them to long term weight outcomes. We adopted a critical realist approach, which posits that it is possible to develop explanatory accounts and models of human behaviour, although these will be situation-dependent and may capture only a small part of the underlying reality [20]. We used Nvivo v11 software (QSR International, Australia) to assist the analysis.

Only the accounts of the 36 participants sampled from the main study were included in the this analysis, which was conducted by LP and SvB. Analysis began with repeated reading of the transcripts and generation of descriptive codes. After independent coding of five baseline transcripts, the researchers met to discuss and iteratively develop a coding framework for analysis of the remaining transcripts. To improve coding consistency and clarity of coding definitions, 10% of the remaining transcripts were coded independently by both researchers, who then met to compare findings, resolve any differences through discussion, and refine coding definitions accordingly.

The analysts then worked together to rearrange and combine the coded concepts into a set of descriptive themes that encompassed the issues most salient to the research question. Transcripts from the second cohort of participants were then incorporated into the analysis. This entailed reading them in temporal sequence for each participant, considering whether the existing coding and thematic frameworks adequately described their content, and specifically looking for examples of congruence or dissonance with themes developed from the first cohort analysis. This process enabled a judgement on whether thematic saturation had been achieved. Within each theme, the analysts also looked for differences in the accounts of those who were more successful in weight management (defined as weight loss maintained at 18 months) compared to those who were less successful (defined as weight gain or no loss at 18 months).

In the final stage of analysis, a set of interpretive themes were developed and an explanatory model was created to link them. The model aimed to provide an explanatory framework linking individual behaviours and experiences to outcomes. The coherence and explanatory power of the model was checked through re-reading transcripts, consulting post-interview field notes and memos created during the analysis, and discussion between the authors. This led to further development of the model to the form presented in this paper.

## Results

Table 1 provides data on the number and duration of interviews conducted at each study stage. Two of the 36 participants who were interviewed at 6 months withdrew from the main study before the final interviews. One recording from the baseline interviews and two from the final interviews were unavailable for transcription due to recorder malfunction, leaving 103 transcripts for analysis.

**Table 1:** Interview samples and durations at each time point

Study stage	Baseline	Post-Programme	Final
**Duration (mins)**	20–30	35–105	20–50
**Participants**	36^a^	36	34

^a^Selected from 100 participants in the main study

Participant group-level demographics and weight change data are provided in Table 2; individualised data is available in Additional file 3.

**Table 2:** Sample demographic and weight change data

Attribute	Value	Number (% of sample)
Sex	Male	14 (39%)
	Female	22 (61%)
Age	18–30	1 (3%)
	31–50	8 (22%)
	51–70	19 (53%)
	70+	8 (22%)
Socioeconomic status (IMD decile) ^a^	1–3	12 (33%)
	4–7	16 (44%)
	8–10	8 (22%)
Ethnicity ^b^	White	36 (100%)
BMI (baseline) kg/m^2^	30–34.9	16 (44%)
	35–39.9	17 (47%)
	40–45.5	3 (8%)
Programme Attendance	≤ 25%	3 (8%)
	26–49%	2 (6%)
	50–80%	11 (31%)
	81–100%	20 (56%)
Weight loss (0–18 months) ^c^	> 10%	6 (17%)
	5–9.9%	6 (17%)
	2–4.9%	7 (19%)
	1.9–0%	3 (8%)
	< 0% (weight gain)	11 (31%)

^a^*IMD* Index of Multiple Deprivation, based on postcode. 1 = lowest level of deprivation

^b^ The mono-ethnicity of the sample reflects that of the wider feasibility study sample

^c^ 18 month weight data were unavailable for 3 participants

### Themes

**Fig. 1:**
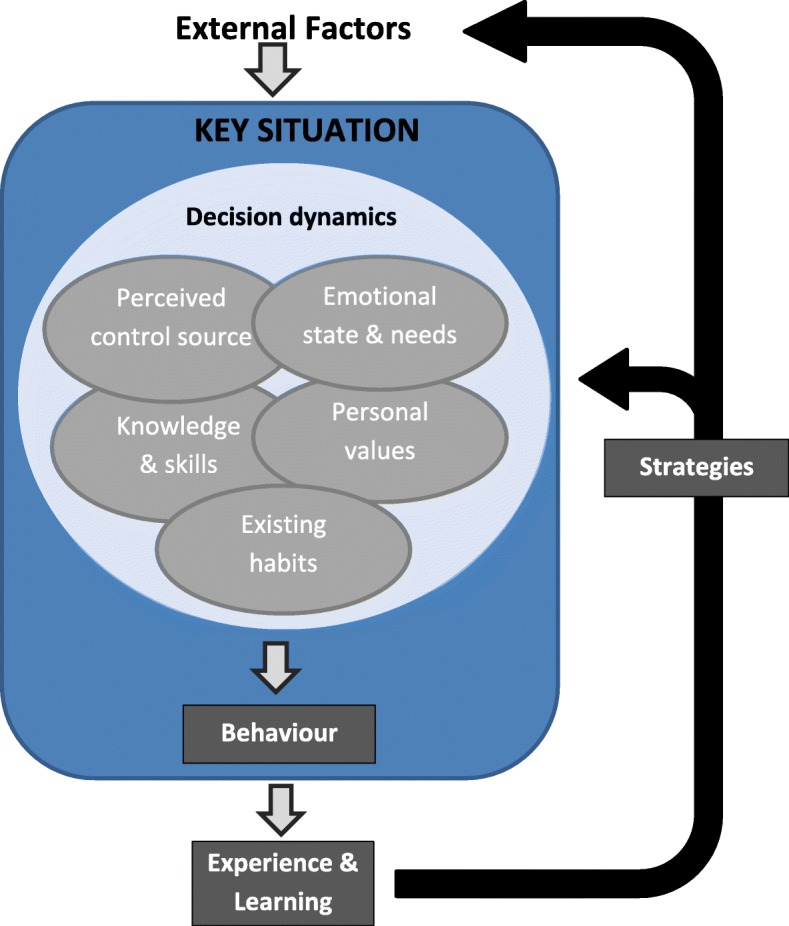
Conceptual model of decision-making and learning in key situations

Our analysis produced several overarching themes that we linked together through a model of in-the-moment decision-making (Fig. 1). The themes were: (1) encountering and managing key situations; (2) the impact of emotion; (3) the source of control; (4) personal values; and (5) acquiring knowledge and skills. In the following sections, each interpretive theme is presented, along with descriptive sub-themes derived during the analysis (which are highlighted in bold). Illustrative quotes are accompanied by brief identifiers of the speaker and interview stage (01 = baseline, 02 = 6 month, 03 = 18 month), their gender (M/F) and an indication of their weight change at 18 months compared to baseline (“++” representing ≥5% loss, “+” representing 0.1–4.9% loss, and “-”meaning no weight loss).

#### Theme 1: encountering and managing key situations

In many accounts, participants described key incidents or situations which, individually or through repetition, significantly impacted their weight management experience and outcomes. Many spoke of facing particular situations that presented strong challenges to the behavioural changes they were making to lose weight. These situations were often set up by external factors such as **social influences**, **work-patterns** or exposure to **food-rich environments**. They could also be attributed to internal experiences such as **emotional arousal**, **boredom** or **fatigue**.

*It’s certain periods of time, like holidays, Christmases, birthday times, where I will go on a binge and be totally unstoppable. [T210–01 F+].*

*There are times I get very low because it’s such a hard job … and it’s at those times I feel that I want to do things I shouldn’t be doing. [T021–02 F-].*

*I can get off to sleep but I then wake a lot and thoughts in my head and everything, and that sometimes is when I think ‘oh I could eat something’. [T201–02 F+].*

Often, key situations evoked a **habitual response**, built up over time, in which an existing obesogenic behaviour automatically followed a regularly encountered cue.

*I slip into bad habits […] I was having two biscuits a day with a cup of tea at 9 o’clock at night and then gradually at two in the afternoon or two in the morning when you’re doing elevenses and then two after lunch and gradually it builds up … Every time you have a cup of tea you have a biscuit. [H008–01 F++].*

*I think I picked up bad habits - when you’re dishing up the kids meals sort of grazing over their meals and what they leave. [T216–01 F-].*

These habitual responses, and the cues that evoked them, were identified by many participants as significant influences on their eating behavioural decisions. The need to break unhealthy habitual responses in key situations was commonly recognised, and those who were most successful spoke of their success in doing so. For instance, they became more aware of these situations and developed **strategies to deal with them**. These included avoiding the situation, managing the external influences operating at the time, or engaging in an inner conversation to encourage a new, healthier response.

*I try not to carry too much money on me as well, because we’ve got a vending machine at work and it’s like a slot machine really. Honestly, I believe that is my biggest reason I’ve lost weight. [G201–02 M++].*

A popular strategy (taught as part of the SkiM programme) was to summon an imagined ‘STOP!’ sign when encountering a key situation, consciously pausing to consider alternative responses or to engage in a distracting activity.

*It was more about a strategy to stop yourself doing the things that you were doing when you were in the moment kind of thing. [T210–02 F+].*

*To stop and think about it for a minute and relax an breathing and just putting it off, and then you have a glass of water, you put it off for ten minutes you probably forget about it. [T006–02 F+].*

Key situations could therefore be seen as moments in which conscious or unconscious behavioural decisions are triggered. Success in weight management involved bringing decision-making into consciousness is such situations to manage them more effectively. Some of those who were successful in the longer term spoke of their new responses to key situations eventually becoming habitual.

#### Theme 2: the impact of emotion

The majority of participants said that their obesogenic behaviours were strongly influenced by their emotional state, and particularly by a desire to alleviate negative feelings or create or enhance positive ones. A number of key situations were identified as influencing decisions about what and when to consume. **Comfort-eating** or drinking was commonly reported, mostly by women but also by some men, although some people said they tended to eat less when they were feeling down. Many also spoke of **eating for pleasure**, or of consuming more in situations where they were feeling happy.

*Emotions play a part in what you do and what you eat. I’d always dive for a dummy in food if I was feeling down, or needed cheering up. And it would be a half packet of biscuits, not just two or three. [G018–02 M-].*

*I love food, I love eating, I love the social experiences around food. [T210–01 F+].*

Some used strategies suggested in the intervention to weaken the link between their emotional state and choosing to eat unhealthily, for instance by deriving equal pleasure from a smaller portion or from healthier food, or by selecting an alternative source of comfort.

*Being able to walk up a hill without getting breathless. That’s where I get the buzz from. So, I haven’t needed to use food to get that buzz and that comfort. I’ve had it in another way. [T201–03 F+].*

However, many felt unable even to attempt this or said they had tried and failed, because nothing else could satisfy the emotional need at the time. Some said they could not think clearly and choose alternative behaviours in situations of strong emotion; others portrayed the decision as both automatic and consciously-willed.

*When I get too overwhelmed with things, I lose that capacity to think straight. [T030–02 F-].*

*It’s something I can chose to do, nobody can tell me, and if I want to go and eat that and I’m feeling worked up or whatever it is, and it almost just automatically happens. [H006–02 F-].*

Emotional responses to progress, or the lack of it, in weight management were also seen by many as driving subsequent behavioural decisions. A commonly-reported example of this was feeling down when self-weighing showed a weight rise or no loss: this could prompt feelings of despair and a decision to stop self-weighing, thereby reducing exposure to this mood-dampening feedback. If a weight management lapse evoked a negative feeling, this could become a key situation for those who were prone to emotional eating, creating a feedback loop that was very difficult to interrupt.

*And then there comes another birthday or Christmas or whatever, and I gain a bit more and I start feeling rubbish about myself, and so begins a massive negative spiral. T210–01 F+].*

On the other hand, emotional responses could also be a positive influence on behavioural decision-making. For example, the pleasure derived from physical exercise or cooking from scratch could help turn these into habitual activities. However, building habits required repeated conscious acts of will in situations where the individual might tend towards making an alternative choice.

*at one stage I thought [the gym] is not going to be for me, I can’t do it. But within […*] *a month or six weeks, I said to my wife ‘I’ve got to go. I really feel the need. I need to get to the gym and do this.’ And it became, again just like your book, a pleasurable activity that I wanted to do. Not a chore, not a pain in the neck. [H005–02 M+].*

In some cases, even negative feelings could prove helpful in decision-making: for instance, a lapse might evoke a sense of annoyance rather than despair, and this became a stimulus to renewed efforts rather than giving up.

#### Theme 3: the source of control

Two distinct narratives were apparent regarding the sources of power, control and **motivation** that participants perceived in their lives. In the ‘out of my control’ narrative, individuals spoke of being swept along by circumstances, and having their choices governed by family responsibilities, work patterns or health issues.

*there’s been some bits of my life, that haven’t been in control because I’ve been focussing in on the work side for example or sorting the family out. [T201–03 F+].*

These individuals often spoke of needing a push from someone else to help them make important decisions, for instance to join the SkiM programme or to stay with the process during challenging situations. They highly valued the sense of accountability that being part of the programme provided, and some expressed disappointment at the perceived lack of surveillance by the facilitators, or the absence of a prescribed eating plan.

*I don’t think hand on heart you can do it on your own. Some people can, I need a lot of encouragement, discipline if you like. [G013–02 M++].*

*I did say to [the programme facilitator] ‘Well, I like you being a policeman’. [T015–02 F+].*

Those who felt controlled by events, or who said they needed others to control them, tended to report fewer behavioural or attitudinal changes. They often identified multiple insurmountable obstacles to approaches and strategies proposed within the programme. For them, behavioural lapses or perceived lack of success often evoked an ‘I just can’t do this’ response at key moments of decision-making. If changes were not sustained, this was often attributed to a loss of the motivation to make the “right” choice that had previously been provided by attendance or by initially-supportive family, friends or colleagues.

*I didn’t have the enthusiasm of people pulling me in the right direction. [G228–02 M++].*

In an alternative ‘I’ve got the power’ narrative, some individuals portrayed themselves as **strong-willed** and self-disciplined, relatively detached from the influence of others and able to control their own situations. They were often pleased that the programme did not provide a set eating plan – although some came to appreciate this omission later on, as it encouraged them to experiment with different approaches and evolve a bespoke plan for themselves. Nevertheless, most experienced weight management as a challenge and emphasised the importance of will-power and self-motivation at key moments. Lapses were experienced but did not knock them off course. They were happy to self-weigh rather than be weighed by the facilitators, and appreciated the programme particularly for the information, advice and problem-solving opportunities it provided.

*I’ve done it for me, I haven’t done it because I thought other people wanted me to do it. [G215–03 F++].*

*the bottom line is you’ve got to do it. And I think too much support is almost as bad as not enough. Because if you become dependent on it when you’re left on your own you’re just going to fail aren’t you? [H005–02 M+].*

Some individuals spoke of an evolving sense of **personal control**, as each experience of success led to greater self-confidence in their ability to make their own choices.

*Instead of being a bit like a mindless eater and drinker I feel as though I’m in a more controlled situation about my own person. [H005–02 M+].*

Most participant accounts fitted one or other of these narrative categories, but the link with personal outcomes was not always apparent. Several individuals expressed confidence in their capacities and felt in control of their lives and choices, yet had poor weight outcomes. Others contrasted the power and self-control they experienced in some areas of life - for instance in their jobs or family roles - with a lack of self-control over their eating behaviour decisions.

#### Theme 4: personal values

Many participants spoke of values that underpinned the choices they made. These could play an important role in the decision to embark on the process of weight management - or to abandon it. They could also influence the day-to-day behavioural choices that were involved in ongoing weight management, for good or ill.

For example, some spoke of the importance of ‘being there’ for partners or children. This could inform the initial decision to lose weight in order to stay healthy and able to look after family; alternatively, it could become a distraction, as concern for others took their attention away from weight management. Some thought it was important to remain functionally independent as they grew older, so they could continue with the activities they enjoyed.

*I did get a bit complacent about it and thought to myself at my age why am I worrying about my weight you know it’s ridiculous. But then I realised you know he [my husband] is quite right. We are beholden to each other to keep ourselves healthy [H008–03 F++].*

Others appeared particularly to value the experience of self-improvement, deriving satisfaction from gaining new skills and confidence in weight management. Several said that seeing things through was important to them, and that this helped them choose to stick with the programme even when they felt alienated from other members of the group, or disconnected from topics being addressed.

Different values could come into conflict when important decisions were at stake. For example, some spoke of deciding that weight loss was not a **priority** after all, because they valued the benefits of their existing behaviours (e.g., pleasure from eating) too highly to change them.

*I’ll be 80 next year, do I have to keep on with this deprivation when you’ve got other things to worry about? [G205–02 F-].*

*So as I see it, the sort of priorities shifted. When you don’t have any other stresses out and about you can focus on yourself a bit, but when other things crop up, really you forget about that so much and you’ve got to reprioritise. [H005–03 M+].*

#### Theme 5: acquiring knowledge and skills

**Gaining knowledge**, particularly of nutrition and healthy eating, was a frequently-cited expectation of the programme and a common theme in post-programme interviews, both for those who were successful and for those who were not. It was often said to underpin change, for example by prompting different decisions in food purchases and meal choices. Some participants who lost little or no weight felt that more specific or detailed knowledge would have made a difference. However, as several recognised, this type of knowledge was important but did not guarantee success.

*It’s energy out that you’ve got to be working on, and energy in you’ve got to be cutting out – and it made a big difference to me, and I should have known about it but it was when it was brought pictorially and together as a discussion within a group, it made a lot of sense. [G239–02 M+].*

*I have a lot of knowledge now and I can’t seem to put it into practice, and that’s what I find really difficult. [T030–02 F-].*

Those who were more successful emphasised the importance **of learning strategies and skills**, whether formally introduced by the programme facilitators or proposed by others in group discussions. Common examples included regular self-weighing, changing the food environment, and communicating needs to friends and family.

*It’s quite good to bounce ideas off of other people and you pick up ideas … and you listen to what others say. [G215–03 F++].*

*What do you want to achieve? Weight, everything I eat. So how am I going to do it? I’ll weigh everything that I eat, so that was one of the first ones. Cook my own food and get my husband to give me smaller portions they were things. How will I cope with any obstacles? If I’m given something I don’t know the calorie count of then I have to adjust everything else and what will I do about it? Adjust, adjust, adjust. So that was at the beginning and then exercise more how will I achieve it? Attend a gym and how will I cope with any obstacles, if I get any pain stop and carry on another day. [H008–02 F++].*

Some participants also said that the programme helped them develop **self-knowledge**, particularly regarding the influences on their choices and behaviours. Such knowledge encouraged some to identify strategies to employ, for instance using assertive communication with friends to co-opt their support. However, several also said that they needed more time or support to develop the capacity to act on insights about deeply ingrained behaviours, or to develop the more complex skills needed to manage social or emotional influences.

*I haven’t fully resolved a lot of issues, and I’ve recognised that’s what I do, and that is half the battle. [G011–02 F-].*

*I understand a year in a change is a time that the body needs to actually settle down and get used to it. And that’s something I’ve only just learnt not long ago and so that is really important. So by that time my habit should be entrenched and there’s much more likelihood of me not falling off the wagon. [H007–02 F++].*

### Linking the themes: the dynamics of decision-making during key situations

We developed a conceptual model of decision-making (Fig. 1) that linked our analytic themes. In this model key situations are set up by particular configurations of personal (physical and psychological) and external (social and environmental) factors. During these situations, individuals make behavioural decisions, consciously or unconsciously. These decisions result from interactions between the influences or personal drivers identified in the thematic descriptions: existing habits, emotional state, perceived power, personal values, knowledge and skills. The decisional outcome, and resulting behaviour, depends on the relative contributions of each of these drivers.

Repetition of behavioural choices in key situations provide experiences and learning that may modify the drivers in key situations. For example, habitual responses may be strengthened or weakened, skills in dealing with the situation may develop, and perceptions of control may change. This process may be consciously enhanced by the development of strategies to modify the key situation or the personal drivers that come into play during these situations.

The prevalence of ‘key situations’ in participant accounts, and the differences in decisional dynamics they describe, suggests that they may be an important determinant of outcomes in weight management. For example, those who were less successful commonly portrayed emotional state and a perceived lack of control as major drivers of their behavioural choices in key situations. While successful weight managers also reported emotions playing a part in their behavioural decisions, they often described them as positive influences, or as outweighed by conscious self-control at those times. Those who were most successful described the development of specific strategies that appeared to change their decisional dynamics in situations that they regularly encountered, for instance by explicitly harnessing their personal values and enhancing their knowledge in ways that would underpin healthy behavioural decision-making.

## Discussion

This study has identified processes that may be important influences on behaviour in weight management, particularly through decision-making during key situations. These situations and the decisional dynamics may differ between individuals and can change over time. Our findings can help to explain why some people are more successful than others, and identify possible targets for change in weight management programmes. In this section, we will briefly relate our findings to existing theory, then explore their implications for practice and future research.

The model we have developed is consistent with several other theories of how thought processes impact upon behaviour in weight management. In particular, ‘dual-process’ approaches to decision-making and behaviour change, which propose that impulsive processes (automatic, habitual, often without conscious awareness) are triggered by situational cues and guide behaviour unless it is actively inhibited or overridden by reflective processes (deliberative, rational, and requiring conscious awareness) [21, 22]. According to dual process theory, repeated reflective management of impulsive processes may permanently modify them [23]. Our model suggests that it may be productive for the individual to reflect on the factors that set up their own key situations, and on the personal drivers of their unconscious decisions at these times. By bringing these dynamics to conscious awareness, personalised targets for change may be identified. Elements of the model also appear in Relapse Prevention Theory [24], which focuses on ‘high risk situations’ that are likely to lead to behavioural lapses and, if repeated, to relapse. The theory highlights the influence of mood and self-efficacy on decision-making in such situations, and the need for coping strategies to manage these factors. The SkiM programme itself was grounded in a theoretical model of psychological ‘tension’ that arises when behaviour change is initiated, and which tends to draw the individual back to their previous behaviours [10]. The tension develops as a result of previous habits, unmet psychological needs, and personal cognitions. Effective weight management requires these sources of tension to be mitigated or managed. Our model is consistent with this understanding, conceptualising moments of ‘high tension’ as key situations, and suggesting that their management is essential to the maintenance of behaviour changes for weight loss.

### Implications

The themes developed in our analysis support the inclusions of several elements of the SkiM programme. Most group sessions began with a review of experience in which participants were encouraged to identify situations they found difficult to manage. These were used as case studies in subsequent discussion of key topics, which included habit-changing, dealing with emotions, and managing social and contextual influences on behaviour. Perceptions of power and personal values, two decisional drivers in the model, were also addressed in the programme during sessions on modifying thoughts, beliefs and self-identity. This study confirms that explicit consideration of specific strategies to manage these drivers during key situations may be helpful. Our findings suggest a three-stage approach that could be incorporated into weight management programmes:

1. identifying key situations that are encountered during the day-to-day experiences of weight management, as well as the social, psychological, physiological and environmental contexts in which they arise;
2. understanding the drivers of decision-making and how they tend to operate during these situations
3. developing and implementing strategies to change the outcomes of the situations through modification or management of their causes, or of the decisional dynamics operating at the time

The accounts we analysed suggest that, although participants often encountered similar key situations, the drivers of behavioural responses to these situations could vary substantially between individuals. This highlights the importance of a personalised approach, involving assessment to identify the drivers operating in key situations for each individual, and the skills and strategies that are most likely to help them. For instance, the dominant influence of emotional status was a common theme among those with poorer outcomes, and so for such individuals the programme may need to put more emphasis on managing its influence.

There are already a number of promising strategies that could be drawn together in a programme that focuses on decision-making in weight management. For example, approaches to habit changing that identify key situations where new habits can be established [25, 26]. Perceptions of personal power may be more challenging to modify, although there is some evidence that self-efficacy for both eating and physical activity behaviours can be enhanced by techniques to break down barriers and gradually building confidence over time [27, 28]. Ways of modifying external factors that initiate some key situations can also be taught. For instance some social pressures may be mitigated by learning and using assertive communication skills. Strategies to optimise personal values in weight management-related decision-making have also been proposed [29]. These may also have some utility in addressing the potent influence of emotions and emotional needs, which accounts in our study suggest can overpower rationality at key times. In any case, the development of approaches to cope with emotional influences should be a priority in weight management research. Other studies have found that emotions and difficulties in emotional regulation are important and under-researched areas in obesity and weight management. For example, a recent review [30] highlighted the presence of emotional functioning difficulties in obesity, and the lack of adaptive strategies among those whose over-eating is emotionally driven. Learning the skills identified here may require more time and resources than are offered in typical weight management programmes in the UK, although targeted modules based on individual needs and using self-help approaches may make this more feasible.

### Study limitations

Our analysis was framed primarily in terms of behavioural science and health psychology, which reflects the backgrounds of the researchers. It gives less emphasis to the influence of physiology and wider socio-economic forces which may also powerfully influence individual capacities and experiences in weight management. It used data from participants in the SkiM programme, whose content may have influenced participants’ perceptions and descriptions of their situations. Therefore our model of decision-making requires validation with participants in other programmes, to assess the transferability of our findings. The mono-ethnicity of our sample (partly a function of the demographics of the recruitment areas), may also have skewed our data and findings. The criterion for “success” at 18 months that we have used is not unproblematic: for some participants whose weight was on upward trajectory before joining the programme, zero weight change could be regarded as a successful outcome. Finally, we recognise that the retrospective nature of the interview involves participants reconstructing and explaining their weight management experiences, and post-hoc rationalisations may have unconsciously influenced their accounts. Using data collected from individuals at several time points means that our model has relevance through the course of the weight management “journey”. The longitudinal nature of the data will be explored in more detail in a subsequent paper.

## Conclusions

This paper presents a model of decision-making in key situations during the process of weight management. The findings have implications for the development of the SkiM programme in particular, and for weight management programmes more generally. By helping individuals to identify the drivers of their decisions in key situations, and equipping them with skills to manage these drivers, programmes may enhance their capacity to sustain the behaviour changes needed for long term weight loss.

## Supplementary information

**Additional file 1.** COREQ checklist for study [17].

**Additional file 2.** Interview topic guides.

**Additional file 3.** Participant data.

## Data Availability

The datasets supporting the conclusions of this article are included within the article and its additional files. Full transcripts of the interviews are not published due to anonymisation issues but requests for such data may be directed to the corresponding author.

## Abbreviations

BMI: Body mass index
GP: General practitioner (Family doctor)
IMD: Index of multiple deprivation
NHS: National Health Service (UK)
SkiM: Skills for weight loss and maintenance (Programme)
