# Supplementary material for: The dynamics of decision-making in weight loss and maintenance: a qualitative enquiry
Source: BMC Public Health. 2020 Apr 28;20:573. doi: 10.1186/s12889-020-08664-y (PMC7189456; doi:10.1186/s12889-020-08664-y)
Supplement: Supplementary file 1 — Additional file 1. COREQ checklist for study [17]. [file 12889_2020_8664_MOESM1_ESM.docx]

**Additional File: COREQ^1^ checklist for study**

| **No. Item** | **Guide questions/description** | **Information** |
| --- | --- | --- |
| **Domain 1: Research team and reﬂexivity** | |  |
| *Personal Characteristics* | |  |
| 1. Interviewer/facilitator | Which author/s conducted the interview or focus group? | Authors 1 and 2 |
| 2. Credentials | What were the researcher’s credentials? E.g. PhD, MD | Author 1: PhD  Author 2: BSc |
| 3. Occupation | What was their occupation at the time of the study? | Author 1: Research Fellow  Author 2: Doctoral Research Associate |
| 4. Gender | Was the researcher male or female? | Author 1: male  Author 2: female |
| 5. Experience and training | What experience or training did the researcher have? | Author 1: formal training in qualitative interviewing and analytical methods, and >10 years’ experience in these activities in >10 studies.  Author 2: formal training in qualitative interviewing and analytical methods, and >5 years’ experience in both these activities in several studies. |
| *Relationship with participants* | |  |
| 6. Relationship established | Was a relationship established prior to study commencement? | No  Before the baseline interview, all participants had previous telephone communication with one of the interviewers, to discuss and screen for participation in the main study, but otherwise they were unknown to the researchers. |
| 7. Participant knowledge of the interviewer | What did the participants know about the researcher? e.g. personal goals, reasons for doing the research | Researchers explained their role in the study as part of the consenting process. |
| 8. Interviewer characteristics | What characteristics were reported about the interviewer/facilitator? e.g. Bias, assumptions, reasons and interests in the research topic | Author 1: age late 50s, healthy BMI, background in physiotherapy and health behaviour change research.  Author 2: age early 30s, health BMI, background in health psychology and health behaviour change research.  Both authors were in the clinically-defined health weight range. |
| **Domain 2: study design** |  |  |
| *Theoretical framework* | |  |
| 9. Methodological orientation and Theory | What methodological orientation was stated to underpin the study? e.g. grounded theory, discourse analysis, ethnography, phenomenology, content analysis | Analysis section |
| *Participant selection* | |  |
| 10. Sampling | How were participants selected? e.g. purposive, convenience, consecutive, snowball | Purposive sub-sample of wider study |
| 11. Method of approach | How were participants approached? e.g. face-to-face, telephone, mail, email | Participants section |
| 12. Sample size | How many participants were in the study? | Results section |
| 13. Non-participation | How many people refused to participate or dropped out? Reasons? | Results section |
| *Setting* | |  |
| 14. Setting of data collection | Where was the data collected? e.g. home, clinic, workplace | Home or university according to participant preference |
| 15. Presence of non-participants | Was anyone else present besides the participants and researchers? | In some cases, a partner or other family member was nearby, but none took part in the interview. |
| 16. Description of sample | What are the important characteristics of the sample? e.g. demographic data, date | Results section and additional file |
| *Data collection* | |  |
| 17. Interview guide | Were questions, prompts, guides provided by the authors? Was it pilot tested? | Semi-structured topic guide with predefined key questions and prompts used; not pilot-tested but refined with experience. |
| 18. Repeat interviews | Were repeat interviews carried out? If yes, how many? | Yes, three interviews over 18 months. |
| 19. Audio/visual recording | Did the research use audio or visual recording to collect the data? | Digital audio-recording |
| 20. Field notes | Were ﬁeld notes made during and/or after the interview or focus group? | Yes |
| 21. Duration | What was the duration of the interviews or focus group? | Interview 1: 20-30 minutes; subsequent interviews 15-50 minutes |
| 22. Data saturation | Was data saturation discussed? | Analysis section |
| 23. Transcripts returned | Were transcripts returned to participants for comment and/or correction? | No |
| **Domain 3: analysis and ﬁndings** | |  |
| *Data analysis* | |  |
| 24. Number of data coders | How many data coders coded the data? | Two; see methods section |
| 25. Description of the coding tree | Did authors provide a description of the coding tree? | No, but available from lead author |
| 26. Derivation of themes | Were themes identiﬁed in advance or derived from the data? | Derived from data |
| 27. Software | What software, if applicable, was used to manage the data? | Nvivo 11 |
| 28. Participant checking | Did participants provide feedback on the ﬁndings? | No |
| *Reporting* | |  |
| 29. Quotations presented | Were participant quotations presented to illustrate the themes/ﬁndings? Was each quotation identiﬁed? e.g. participant number | Yes. See Results section |
| 30. Data and ﬁndings consistent | Was there consistency between the data presented and the ﬁndings? | Results section |
| 31. Clarity of major themes | Were major themes clearly presented in the ﬁndings? | Results section |
| 32. Clarity of minor themes | Is there a description of diverse cases or discussion of minor themes? | Yes. See Results section |

1. Developed from: Tong A, Sainsbury P, Craig J. Consolidated criteria for reporting qualitative research (COREQ): a 32-item checklist for interviews and focus groups. *International Journal for Quality in Health Care*. 2007. Volume 19, Number 6: pp. 349 – 357
