# Supplementary material for: The dynamics of decision-making in weight loss and maintenance: a qualitative enquiry
Source: BMC Public Health. 2020 Apr 28;20:573. doi: 10.1186/s12889-020-08664-y (PMC7189456; doi:10.1186/s12889-020-08664-y)
Supplement: Supplementary file 2 — Additional file 2. Interview topic guides. [file 12889_2020_8664_MOESM2_ESM.docx]

**Supplementary material: Interview topic guides**

These are the final topic guides used for baseline and follow-up interviews. They contain all the questions that were posed at the interviews, including several on feasibility issues that are not dealt with in this paper.

**topic guide (questions and prompts) for baseline interview**

| Did you complete the “my weight journey” picture we asked you to draw? If not, no problem, we will just talk about some of the issues it is meant to raise   - Ask the respondent to explain its main features. - Focus on main influences at times of weight change |
| --- |
| Why do you want to lose weight now? |
| If you have tried to control your weight in the past, what has helped you do this?   - Things you have done or changed to lose weight or to stop it increasing - Other people, organisations, influences - How have they been helpful? - What are the biggest challenges for you in controlling your weight? |
| If you regained lost weight in the past, why do you think this happened?   - Were there things about yourself ? - Where there things about the people or world around you? |
| How do you feel currently about managing your weight?   - Is it something you feel particularly positive or negative about – why? |
| Why have you decided to take part in this weight management programme?   - What has prompted you to join it? - What are you expecting will happen? - What do you hope to get out of it? |
| Are there people around you who could help or hinder your weight management   - Living alone/with others - Friends and colleagues |

**topic guide (questions and prompts) for post-programme interview**

| **Part 1: weight management experiences** |
| --- |
| How has your weight management gone over the last six months?   - How do you feel about your progress? - What have been your main achievements? - What has helped you most? - What has been most difficult? Why? - Do any particular experiences stand out for you? Why? |
| What were the main changes you made to try to lose weight?   - What worked well for you (eating / PA) - What didn’t work so well – why? |
| Have you noticed any changes in yourself over the last six months   - What were these? Physical/psychological - What has been your most important learning - What do you do differently now? |
| Have you used any of the strategies suggested in the programme?   - If so, how did it go? - Examples: impulse control, managing stress/mood, changing your thinking, changing your habits, negotiating with other people, planning, self-monitoring, goal setting, dealing with lapses, finding other ways of meeting your needs, changing your food environment |
| Do you see yourself or weight management differently in any way now?   - E.g. more confident, in control, positive, skilled - more active, healthier eater - WM as lifelong, not a “diet” - Flexible approach - New habits |
| What are the main challenges in weight management for you still?   - Do you experience “tension”, drawing you back to previous ways of doing things? |
| How do you see your weight management going in the future?   - Plans / goals? - Will you be able to keep up the changes you have made? - Optimistic? Motivated? - Sources of support / motivation |
| **Part 2: Views on SkiM programme and study** |
| How was the programme for you?   - What did you find most helpful, why? - Were there things you found unhelpful? What/why? - Were there things that were difficult but still helpful? - What did you enjoy most? - How did it compare to what you expected? - Was anything missing that you would have found helpful? |
| Which bits of the programme influenced what you did / how you changed?   - Why? - What strategies or ideas have you taken on board? - What did you learn from it? |
| What did you think of the Facilitators?   - Were there things you found particularly helpful in the way they did things? - Were there things you found particularly unhelpful in the way they did things? - How did they handle the group? - How would you describe their way of working with you (e.g. professional, caring, directive, collaborative) - Did you feel confident in them? Why (not)? - How much did the facilitator listen to you and take your own needs and preferences into account? - Is there any way you think we could improve the way the Facilitators work in this programme? |
| How was it, being part of a group?   - Was it helpful / unhelpful for your needs? - Did the group work well together? |
| - (Provider B) – how did you find the initial 1:1 meeting with the Facilitator? |
| Did anything outside the programme affect your weight management   - E.g. slimming clubs, family circumstances, work, illness, events |
| What did you think of the different resources that were available?   - Skim folder and handouts - Website - (Provider B) gym - Pedometer – was it motivational? - Texting |
| How were the practicalities of the programme and venue for you?   - Distance to travel - Venue comfort, suitability, facilities - Programme timing, frequency, session duration |
| If we were to run the programme again, would you change anything?   - What would you prioritise? - What would you drop? |
| How was your experience of taking part in the research?   - Were there any problems? If so, what - Communication with the research team - Questionnaires - Anything we could do to make it better? |
| Is there anything else you would like to say about your weight management or taking part in the SkiM study? |

**topic guide (questions and prompts) for 18-month interview**

| **Part 1: weight management experiences** |
| --- |
| Please tell me about how your weight management has gone since the end of the SkiM programme (refer to weight journey picture the participant was asked to complete before the interview)   - Are you still trying to manage your weight? - What has gone well - What have been the main influences on your weight over the period? - What has helped you continue to manage your weight? - What have been the challenges in continuing to manage your weight - Have you been getting support for your weight management from any source? Explain. |
| What have been your main strategies for maintaining your weight loss or continuing to lose weight? Tell me how that has gone.   - Prompts: Impulses, STOP, urge surfing; Trying to make/break a habit; Dealing with other people; Managing feelings, stress, negative thoughts; Coping with slips and lapses; Self-weighing, planning. |
| How do you see yourself now, compared to 18 months ago before you came into this study?   - Any changes – physical/psychological/social/behavioural - Changes in what’s important to you? - Finding other ways of meeting your needs - Doing weight management automatically - A different lifestyle - Describe yourself then and now |
| Show the SkiM model of tension and ask   - Do you remember this at all? - Does it describe your situation at all? - Which bits are most relevant to you? |
| How do you feel about managing your weight now and in the future?   - Optimism / confidence /sense of tension or struggle |
| **Part 2: Views on SkiM programme and study** |
| Looking back at the SkiM programme, what are your thoughts on it now?   - If you were to describe it to someone else, what would you say? - What were its good and bad points? |
| Are there any bits of it that you particularly remember   - Were they useful, are you still using them? |
| Have you continued to use any of the SkiM resources?   - KiO book - Website - Progress tracker - Programme folder / handouts |
| How was your experience of taking part in the research?   - Were there any problems? If so, what - Communication with the research team - Questionnaires - Anything we could have done to make it better? - Did the vouchers we offered for taking part in assessments make any difference to your willingness to do them? |
| Is there anything else you would like to say about your weight management or taking part in the SkiM study? |
