# Supplementary material for: The dynamics of decision-making in weight loss and maintenance: a qualitative enquiry
Source: BMC Public Health. 2020 Apr 28;20:573. doi: 10.1186/s12889-020-08664-y (PMC7189456; doi:10.1186/s12889-020-08664-y)
Supplement: Supplementary file 3 — Additional file 3. Participant data. [file 12889_2020_8664_MOESM3_ESM.docx]

| **ID** | **Sex** | **Age**  **(y)** | **Education**  **level** | **Employment**  **status** | **IMD  Decile** | **BMI Baseline**  **(kg/m^2^)** | **Programme  attendance** | **6M weight change** | **18M weight change** | **Success** |
| --- | --- | --- | --- | --- | --- | --- | --- | --- | --- | --- |
| G001 | F | 51-70 | 5 | 6 | 3 | 35 | 88% | -6% | +3% | - |
| G004 | F | 51-70 | 5 | 6 | 3 | 32 | 25% | +1% | -2% | + |
| G011 | F | 51-70 | 6 | 6 | 7 | 34 | 100% | -3% | +4% | - |
| G013 | M | 51-70 | 5 | 6 | 7 | 43 | 100% | -8% | -9% | ++ |
| G018 | M | >70 | 5 | 6 | 3 | 36 | 100% | -1% | +12% | - |
| G201 | M | 51-70 | 5 | 1 | 10 | 34 | 100% | -4% | -10% | ++ |
| G205 | F | >70 | 4 | 6 | 8 | 31 | 100% | -7% | 0% | - |
| G207 | F | 51-70 | 2 | 1 | 8 | 42 | 92% | -5% | -3% | + |
| G215 | F | >70 | 5 | 6 | 6 | 31 | 85% | -11% | -15% | ++ |
| G220 | M | 51-70 | 5 | 1 | 10 | 39 | 67% | +3% | +4% | - |
| G223 | M | 31-50 | 6 | 1 | 3 | 39 | 83% | -5% | -4%* | + |
| G228 | M | >70 | 3 | 6 | 7 | 30 | 100% | -7% | -7% | ++ |
| G230 | M | 31-50 | 7 | 7 | 10 | 34 | 25% | +1% | -4% | + |
| G235 | M | 51-70 | 7 | 1 | 6 | 34 | 77% | +1% |  | ? |
| G239 | M | 51-70 | 7 | 7 | 8 | 36 | 75% | -6% | -3% | + |
| G244 | F | >70 | 7 | 6 | 3 | 38 | 100% | 0% | +5% | - |
| H005 | M | 51-70 | 5 | 6 | 8 | 34 | 88% | -4% | -3% | + |
| H006 | F | 31-50 | 6 | 1 | 5 | 37 | 50% | +6% | +6% | - |
| H007 | F | >70 | 7 | 6 | 9 | 36 | 75% | -29% | -32% | ++ |
| H008 | F | 51-70 | 5 | 6 | 4 | 35 | 100% | -11% | -18% | ++ |
| T006 | F | 51-70 | 5 | 1 | 6 | 33 | 93% | -7% | -4% | + |
| T013 | F | >70 | 5 | 5 | 2 | 33 | 64% | -4% | 0% | - |
| T015 | F | >70 | 5 | 6 | 3 | 35 | 93% | -6% | -13%* | + |
| T021 | F | 51-70 | 5 |  | 5 | 38 | 64% | 0% | +10% | - |
| T027 | F | 51-70 | 5 | 1 | 5 | 34 | 71% | -7% | +1% | - |
| T030 | F | 31-50 | 7 | 1 | 1 | 37 | 14% | +1% | +4% | - |
| T032 | F | 18-30 | 4 | 7 | 1 | 40 | 79% | -20% | -30% | + |
| T201 | F | 51-70 | 7 | 7 | 7 | 38 | 85% | -22% | -28% | + |
| T208 | F | 51-70 | 5 | 2 | 4 | 38 | 46% | +4% | +3% | - |
| T210 | F | 31-50 | 7 | 1 | 4 | 36 | 54% | -3% | -3% | + |
| T213 | F | 31-50 | 7 | 1 | 6 | 34 | 31% | 0% | -1% | + |
| T216 | F | 31-50 | 5 | 1 | 6 | 35 | 91% | -4% | +1% | - |
| T229 | M | 31-50 | 6 | 1 | 6 | 34 | 100% | -8% | -6% | + |
| T230 | M | 51-70 | 3 | 1 | 1 | 46 | 92% | -12% | -9% | ++ |
| T233 | M | 51-70 | 7 | 1 | 2 | 32 | 64% | +1% | -7% | ++ |
| T236 | M | 51-70 | 5 | 1 | 1 | 38 | 91% | -17% | -8% | + |

Notes

**Education level**

1. Primary school
2. Some secondary school
3. Secondary school up to 16 years
4. Secondary school up to 18 years
5. Additional training
6. Undergraduate university
7. Postgraduate university

**Employment status**

1. Paid or self-employed
2. Voluntary employment
3. Unemployed
4. Student
5. Housewife/husband/family carer
6. Retired

**IMD Decile**

The Index of multiple deprivation is a measure of relative deprivation used to rank neighbourhoods across the UK, based on postcode. It takes into account factors such as income, education, employment, housing, crime and health. Then higher the decile, the higher the level of deprivation in that locality compared to other localities.

**SkiM Programme Attendance**

Figure represents the number of group sessions attended by the individual as a percentage of the sessions offered in the programme.

**Weight change**

- Positive = weight gain, negative = weight loss compared to baseline
- 18M data marked with asterisks are for 12 months because 18 month data unavailable
- One 18M data point missing as both 12 and 18 month data unavailable

**Success**

Success in weight management was defined by percentage weight change at 18 months compared to baseline:

++ ≥ 5% loss

+ 0.1-4.9% loss

- No loss
